# Retrospective review using targeted deep sequencing reveals mutational differences between gastroesophageal junction and gastric carcinomas

**DOI:** 10.1186/s12885-015-1021-7

**Published:** 2015-02-06

**Authors:** Hector H Li-Chang, Katayoon Kasaian, Ying Ng, Amy Lum, Esther Kong, Howard Lim, Steven JM Jones, David G Huntsman, David F Schaeffer, Stephen Yip

**Affiliations:** 1 University of British Columbia, Vancouver, Canada; 2 Division of Anatomic Pathology, Department of Pathology and Laboratory Medicine, Vancouver General Hospital, 855 12 Ave W, Vancouver, BC V5Z 1 M9 Canada; 3 Department of Molecular Oncology, British Columbia Cancer Agency, Vancouver, Canada; 4 Canada’s Michael Smith Genome Sciences Centre, British Columbia Cancer Agency, Vancouver, Canada; 5 Centre for Translational and Applied Genomics, British Columbia Cancer Agency, Vancouver, Canada; 6 Department of Medical Oncology, British Columbia Cancer Agency, Vancouver, Canada

**Keywords:** Gastric cancer, Gastroesophageal junction cancer, Gastric cancer genomics, Gastric cancer sequencing

## Abstract

**Background:**

Adenocarcinomas of both the gastroesophageal junction and stomach are molecularly complex, but differ with respect to epidemiology, etiology and survival. There are few data directly comparing the frequencies of single nucleotide mutations in cancer-related genes between the two sites. Sequencing of targeted gene panels may be useful in uncovering multiple genomic aberrations using a single test.

**Methods:**

DNA from 92 gastroesophageal junction and 75 gastric adenocarcinoma resection specimens was extracted from formalin-fixed paraffin-embedded tissue. Targeted deep sequencing of 46 cancer-related genes was performed through emulsion PCR followed by semiconductor-based sequencing. Gastroesophageal junction and gastric carcinomas were contrasted with respect to mutational profiles, immunohistochemistry and *in situ* hybridization, as well as corresponding clinicopathologic data.

**Results:**

Gastroesophageal junction carcinomas were associated with younger age, more frequent intestinal-type histology, more frequent p53 overexpression, and worse disease-free survival on multivariable analysis. Among all cases, 145 mutations were detected in 31 genes. *TP53* mutations were the most common abnormality detected, and were more common in gastroesophageal junction carcinomas (42% vs. 27%, p = 0.036). Mutations in the Wnt pathway components *APC* and *CTNNB1* were more common among gastric carcinomas (16% vs. 3%, p = 0.006), and gastric carcinomas were more likely to have ≥3 driver mutations detected (11% vs. 2%, p = 0.044). Twenty percent of cases had potentially actionable mutations identified. R132H and R132C missense mutations in the *IDH1* gene were observed, and are the first reported mutations of their kind in gastric carcinoma.

**Conclusions:**

Panel sequencing of routine pathology material can yield mutational information on several driver genes, including some for which targeted therapies are available. Differing rates of mutations and clinicopathologic differences support a distinction between adenocarcinomas that arise in the gastroesophageal junction and those that arise in the stomach proper.

**Electronic supplementary material:**

The online version of this article (doi:10.1186/s12885-015-1021-7) contains supplementary material, which is available to authorized users.

## Background

Gastric cancer accounts for over 10,000 deaths annually in the United States [1], and is the second most common cause of cancer mortality worldwide [2]. Although carcinomas of the gastroesophageal junction (GEJ) have been grouped with gastric carcinomas in cancer registries and in clinical trials for targeted therapies [3], lesions at these two sites have distinct clinical features. Adenocarcinomas of the stomach proper are primarily caused by *Helicobacter pylori* infection [4] and are decreasing in incidence worldwide [1]. In contrast, GEJ cancers are most associated with gastroesophageal reflux disease [2-5] and obesity [6], and the incidence of GEJ carcinomas has remained stable over the past 20 years [7]. In addition, the prognosis of GEJ carcinomas has been noted to be worse than gastric carcinomas, and there is uncertainty as to whether GEJ carcinomas should be staged as gastric or esophageal tumors [8]. Recognizing the distinction between carcinomas of the GEJ, esophagus, and stomach may enhance the collection of meaningful epidemiologic data and result in increased management precision [9].

Several studies have noted differences in the molecular characteristics of GEJ carcinomas versus those that arise elsewhere in the stomach. *TP53* mutations are more frequent in the GEJ than in the distal stomach, while loss of heterozygosity of the *TP53* locus is also more common in GEJ tumors [10,11]. Significant differences in promoter methylation rates of *APC* and *CDKN2A* have also been described [12]. Furthermore, differences in *APC* mutation rates and protein expression, as well as differences in global gene expression profiles between the two sites have also been demonstrated [13-16].

Testing of amplifications of the *ERBB2* (also known as *HER2*) gene in gastric and gastroesophageal junction cancers is now routine practice in many institutions [17]. Similarly, testing for driver mutations, particularly single nucleotide substitutions, in oncogenes and tumour suppressor genes currently informs treatment in adenocarcinomas of other sites such as the lung and colon [18-20]. As further molecular targets are discovered across disease sites, effective assays will be required to determine cancers’ susceptibility to targeted treatment.

Next-generation sequencing may be used in the near future to interrogate multiple genes in a single sample, and these data could be used to inform clinicians of driver mutations and guide targeted treatment. Targeted panel sequencing is a form of next-generation sequencing where single nucleotide variants are detected in a limited number of previously determined genomic loci, which by intention are often prognostically and therapeutically critical. Panel sequencing enables multiplexing of samples, and deep coverage (>500x) facilitates the analysis of suboptimal template material from archival tissue and samples with low tumor cellularity. The narrower set of genes also allows for quicker specimen processing and bioinformatic analysis. Thus, actionable results can be obtained within days, rather than the weeks, compared to whole genome and exome approaches. However, data is restricted by the inherently biased selection of genes, and the inability to detect copy number changes, loss of heterozygosity, and structural rearrangements such as gene fusions. Thus, the effective use of NGS requires careful assessment of technologies, assay limitations, template requirements, and the research and clinical questions under consideration.

The objectives of this study were to probe the utility of panel sequencing on formalin-fixed paraffin-embedded (FFPE) tissue, and to compare clinically annotated GEJ and gastric carcinomas through panel sequencing of the hotspots of 46 cancer genes. We also sought to compare the frequencies of mutations identified with panel sequencing of hotspots against whole-exome sequencing, using publically available data from The Cancer Genome Atlas.

## Methods

### Case selection and retrieval of clinicopathologic data

Institutional ethics approval was obtained from the University of British Columbia/British Columbia Cancer Agency research ethics board (#H07-2807), and research was conducted in accordance with the Helsinki declaration. Cases of gastric carcinoma were retrieved from departmental archives from the British Columbia Cancer Agency (BCCA), a provincial referral center. Inclusion criteria were referral to the agency between 2004 and 2010, available FFPE tissue from surgical resection of the primary tumor, complete clinicopathologic data including clinical outcomes on follow-up, and the absence of metastatic disease at presentation. Biopsy specimens of primary and metastatic lesions were excluded due to the absence of complete pathologic data. GEJ location was defined as lesions with an epicenter within 5 cm of the proximal end of the gastric rugal folds [21]. No distinction was made between tumors with regards to the location of their epicenter within the 5 cm of the GEJ (i.e. Siewert type was not recorded) [22]. Carcinomas located exclusively within the esophagus were excluded, as per the most recent WHO criteria [21]. All gastric tumors located distal to the GEJ were binned together for this study. Clinicopathologic data was collected retrospectively through review of patients’ charts by a member of the clinical team, as well as through review of pathology reports.

### Tissue microarray construction, immunohistochemistry and *in situ* hybridization

Tissue microarray construction was carried out using two 0.6 mm cores from two separate sections of tumor. Immunohistochemical staining for p53 (1:100; clone DO-7, Ventana Medical Systems, Tucson, AZ), Baf250a (1:75; Sigma-Aldrich, St. Louis, MO), and the mismatch repair (MMR) proteins including hMLH1 (1:25; clone ES05, Leica, Wetzlar, Germany), MSH2 (1:5; clone 25D12, Leica), hMSH6 (1:300; clone PU29, Leica), and hPMS2 (1:150; clone MOR4G, Leica) was performed on the XT platform (Ventana). Expression of p53 was scored as absent (<1% nuclear staining), normal (1-60% nuclear staining of any intensity), or overexpression (>60% nuclear staining of any intensity). Baf250a and MMR proteins were scored as intact (≥1% staining) or negative (<1% staining) based on protein expression specifically in tumour cells (i.e. immune and stromal expression was ignored). *ERBB2* silver *in situ* hybridization (SISH) was performed using the XT automatic IHC/ISH staining platform (Ventana). A *ERBB2*:CEP17 ratio <2.0 was classified as non-amplified, and a value ≥2.0 as amplified. Enumeration of SISH signals was based on established protocols [17].

### DNA sample processing, sequencing, and variant calling

In each case, hematoxylin and eosin slides were used to guide macrodissection or scrolling of tumor tissue from FFPE slides following outlining of tumours by an anatomical pathologist. Tumor DNA from each case was extracted using Qiagen FFPE DNA extraction kit (Qiagen, Venlo, Netherlands); no germline DNA was extracted. Extracted DNA was quantified using the QUBIT HS dsDNA assay (Life Technologies Gaithersburg, MD, USA); all cases had a minimum of 10 ng of DNA extracted from FFPE, in keeping with a previously reported requirement for the assay [21]. A minimum A260/280 ratio of 1.8 was required for each DNA sample. DNA amplicon library construction was performed using DNA primers from the Ion Ampliseq™ Cancer Hotspot Panel v1 (Life Technologies). The kit consists of 207 primer pairs that cover 739 hotspots within 46 cancer-related genes (Additional file 1: Table S1). Indexed amplicon libraries were pooled for emulsion polymerase chain reaction and sequencing on the Ion Torrent PGM platform (Life Technologies). A minimum of at least 500x base pair coverage was required for each case. Variant calling was performed using the Torrent Variant Caller v2.2 (Life Technologies) using the hg19 reference genome. Only variants present at frequencies ≥5% were considered. Because germline DNA was unavailable for comparison, variants were excluded as possible somatic mutations if they were identified as single nucleotide polymorphisms with mean allele frequencies of >0 within the dbSNP database (www.ncbi.nlm.nih.gov/SNP); their status as non-germline variants was further confirmed using a PubMed search (www.ncbi.nlm.nih.gov/pubmed).

### Comparison with the Cancer Genome Atlas (TCGA) data

Curated somatic mutation calls for 281 TCGA stomach adenocarcinoma samples with known anatomical sites were retrieved from the TCGA Data Portal (https://tcga-data.nci.nih.gov/tcga/) on February 19, 2014. Protein-coding mutations located in the regions amplified by the Ion Ampliseq™ Cancer Hotspot Panel v1 in each of the 46 genes were obtained for cases and stratified by location (60 cardia/proximal and gastroesophageal junction *versus* 221 fundus/body, antrum/distal and stomach NOS). Copy number data, RNA expression data, and protein expression data were not considered as our own assay only detects single nucleotide variants (SNVs) and small basepair insertions/deletions (INDELs). The frequencies of mutations, irrespective of the type of mutation, were compared versus the hotspot multiple panel sequencing that we performed.

### Data analysis

Mann–Whitney U-tests and student t-tests were used to compare linear variables, where appropriate. Fisher exact and chi-square tests, where appropriate, were used to compare categorical values. Survival analyses were performed using log-rank (Kaplan-Meier) and Cox proportional hazards tests. The 46 panel genes were mapped to the Kyoto Encyclopedia of Genes and Genomes (KEGG) [22,23] and the Ingenuity® Integrated Pathway Analysis program (Qiagen) to identify oncogenic pathways and networks enriched for mutations, and to test for statistically significant differences between gastroesophageal junction and gastric adenocarcinoma specimens. *P* values were corrected for multiple testing using the Benjamini–Hochberg (BH) correction [24]. All statistical tests were two-tailed and a *P* value of < .05 was considered statistically significant. Statistical analyses were performed using SPSS Statistics software (v22, IBM, Armonk, NJ, USA) and the R statistical language v.2.15.1 (R Core Team (2012). R: A language and environment for statistical computing. R Foundation for Statistical Computing, Vienna, Austria. ISBN 3-900051-07-0, URL http://www.R-project.org/).

## Results

**Figure 1:**
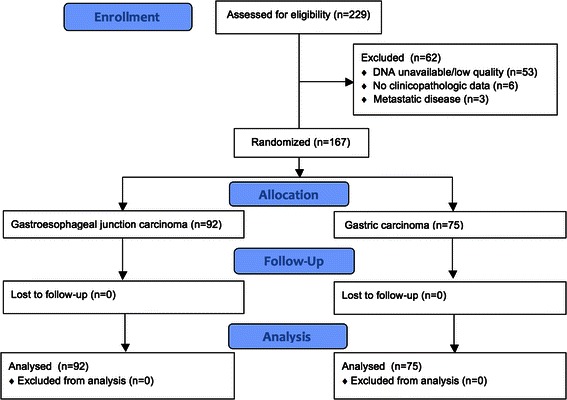
**Flow diagram detailing case selection and exclusion for the study cohort.**

Within departmental archives at the BCCA, 229 resection specimens of gastric and GEJ carcinomas were obtained from 2004 to 2010 and were available for construction of a tissue microarray. DNA was available for extraction for 176 cases. No clinicopathologic data was available for correlation in 6 cases. Three cases had metastatic disease documented within a month of presentation, and these were excluded from the analysis. Of the remaining 167 cases, 92 originated in the gastroesophageal junction and 75 originated in the remainder of the stomach (Figure 1).

### Clinicopathologic differences between GEJ and gastric carcinomas

**Figure 2:**
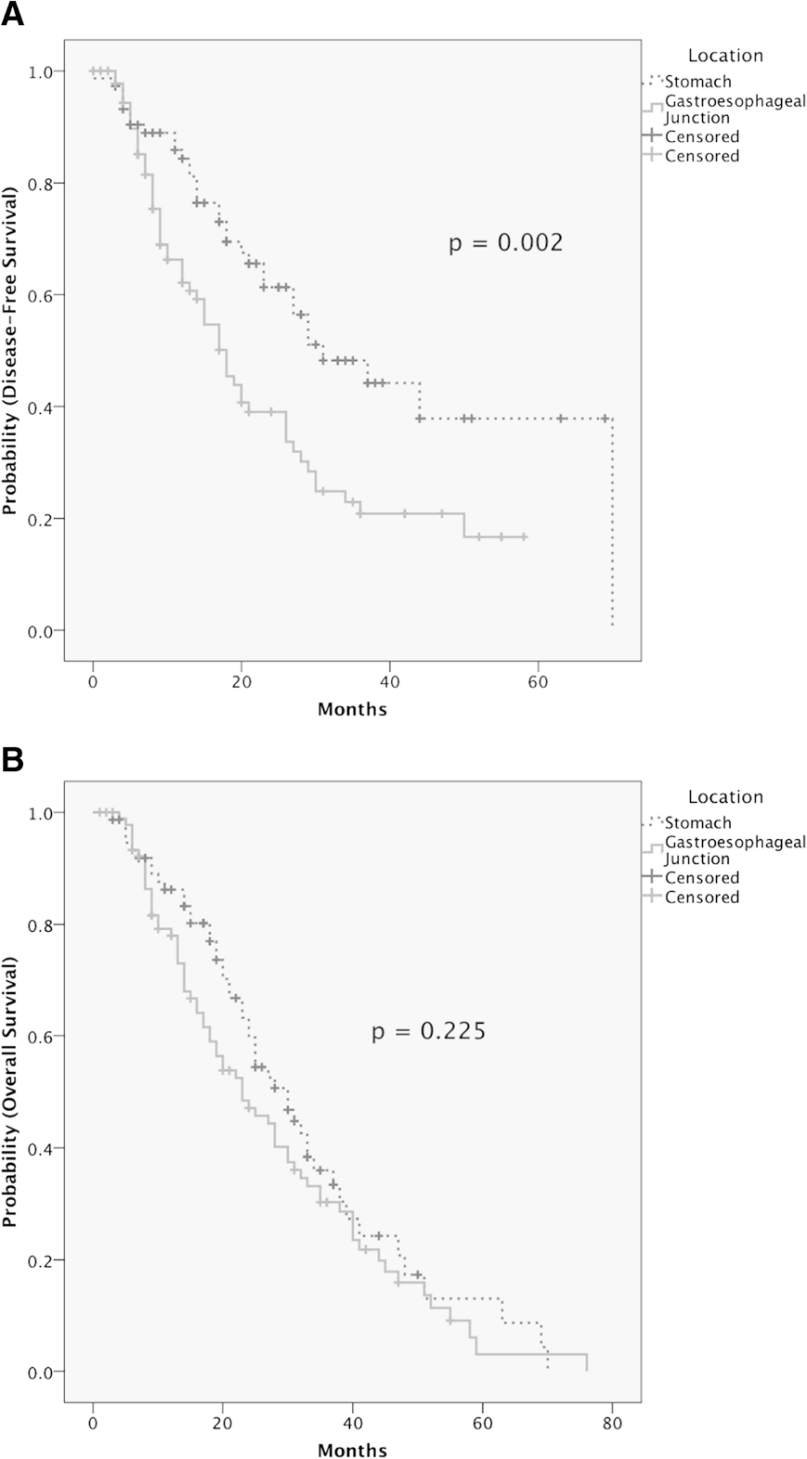
**Comparison of disease-free survival and overall survival between patients with gastroesophageal and gastric carcinomas. A)** Disease free survival was significantly worse for gastroesophageal carcinomas (solid lines) compared to gastric carcinoms (dotted lines), Log-rank test; p = 0.002, though **B)** overall survival did not differ between the two disease sites (Log-rank test; p = 0.225).

The clinicopathologic features of these cases are summarized in Table 1 and anonymized clinical data is provided in a supplemental file (Additional file 2: Table S2). GEJ carcinomas were associated with younger age at resection, more frequent intestinal-type and less frequent diffuse histology, more frequent p53 overexpression and less frequent loss of p53 expression, more frequent stage III disease, less frequent stage I disease, and more frequent recurrences. Disease-free survival was significantly worse among patients with GEJ carcinomas (Figure 2A), though the two cohorts were not statistically different in terms of overall survival (Figure 2B). Other clinicopathologic features were similar between tumors of the two locations, including T-stage, resection margin involvement, *ERBB2* amplification, and MMR protein loss (Table 1). The proportion of diffuse carcinomas in the Lauren classification) was similar between the two sites. Subgroup analysis of only intestinal-type carcinomas showed persistent differences between GEJ and gastric carcinomas in disease-free survival and p53 expression. Differences in age, p53 expression and outcome persisted when considering only intestinal-type carcinomas, as well as when tumours were stratified into three subtypes (proximal non-diffuse, diffuse, and distal non-diffuse) as suggested by Shah et al. [16] (Additional file 3: Table S3).

**Table 1:** **Summary of the clinocopathologic variables in the cohort’s clinicopathologic variables within cardia and non-cardia adenocarcinomas**

Clinicopathologic variable	Gastroesophageal junction (n = 92)	Non-cardia (n = 75)	Overall (n = 167)	p
**Age (mean, years)**	61.5 +/- 9.6 [33-80]	66.3 +/- 11.9 [33-84]	63.7 +/- 10.9 [33-84]	**0.001**
**Sex**				0.297
Male	70 (76)	51 (68)	121 (73)	
Female	22 (24)	24 (32)	46 (27)	
**Histologic subtype (Lauren)**				**0.008**
Intestinal	65 (71)	36 (48)	101 (60)	
Diffuse	15 (16)	26 (35)	41 (25)	
Mixed	12 (13)	13 (17)	25 (15)	
**Stage (AJCC)**				**0.031**
IA-B	10 (11)	19 (25)	29 (17)	
IIA-B	60 (65)	45 (60)	105 (63)	
III-A-C	22 (24)	11 (15)	33 (20)	
**Grade**				0.415
Well differentiated (G1)		4 (5)	10 (6)	
Moderately differentiated (G2)	39 (42)	25 (33)	64 (38)	
Poorly differentiated (G3)	47 (51)	46 (61)	93 (56)	
**Resection margin**				0.306
Uninvolved	74 (80)	65 (87)	139 (83)	
Involved	18 (20)	10 (13)	28 (17)	
***ERBB2*** **amplification**				0.654
Absent	78 (85)	66 (88)	144 (86)	
Present	14 (15)	9 (12)	23 (14)	
**BAF250a (** ***ARID1A*** **) expression**				0.111
Intact	73 (79)	51 (68)	124 (74)	
Absent	19 (21)	24 (32)	43 (26)	
**p53 expression**				**2.8x10** ^**-4**^
0 – absent	32 (35)	47 (63)	79 (47)	
1 – normal (1-60%)	17 (19)	14 (19)	31 (19)	
2 - increased (>60%)	43 (47)	14 (19)	57 (34)	
**Mismatch repair proteins**				0.244
Intact	77 (84)	57 (76)	135 (80)	
Abnormal	15 (16)	18 (24)	33 (20)	
**Number of recurrences**	57 (62)	32 (43)	89 (53)	**0.019**
**Median progression-free survival (Months)**	12	18	15	
**Number of deaths**	69 (75)	48 (64)	117 (70)	0.130
**Median overall survival (Months)**	18.0	23.0	20.0

On multivariable analysis, GEJ location was independently associated with worse disease-free survival (Cox Proportional Hazard =2.08 [95% confidence interval: 1.25-3.44], p = 0.005) along with margin status and microsatellite instability (Additional file 4: Table S4). Age, tumor grade and margin involvement were independently prognostic of overall survival (Additional file 5: Table S5).

### Mutations identified with the cancer panel

**Figure 3:**
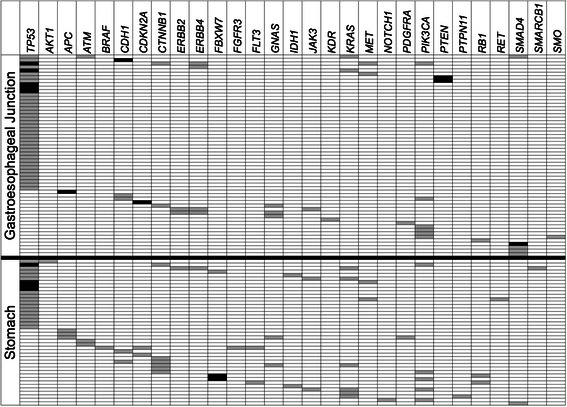
**Somatic mutations identified in gastroesophageal junction and gastric carcinomas.***TP53* mutations were identified in a larger proportion of gastroesophageal junction tumors, while abnormalities in *APC*/*CTNNB1* occurred more frequently in gastric tumors. Black blocks represent truncating mutations, while grey blocks represent missense mutations. Cases and genes in which mutations were not identified are not included.

Among all cases, 145 mutations were detected in 31 genes, with 75 mutations detected among 57 of the tumors from the GEJ, and 70 mutations detected among 43 gastric tumors (Figure 3). No mutations were detected in 35 (38%) and 32 (43%) of the tumors from the GEJ and stomach, respectively. *TP53* was the most commonly mutated genes, with variants identified in 59 of 167 cases (35%). The next most commonly mutated genes were *PI3KCA* (6%), *CTNNB1* (5%), *KRAS* (5%) and *SMAD4* (4%). Other variants included hotspot mutations in *IDH1* (2 cases), *JAK3* (3 cases), and *FLT3* (2 cases). A single mutation was identified in 70 cases (42%), 2 mutations were identified in 20 cases (12%), and ≥3 mutations were identified in 10 cases (6%).

No mutations were identified within the hotspot regions of *ALK*, *CSF1R*, *EGFR*, *FGFR2*, *HNF1A*, *HRAS*, *JAK2*, *MPL*, *NPM1*, *NRAS*, *SRC*, *STK11* or *VHL*. All variant calls are available in the supplementary data (Additional file 6: Table S6).

### Differences in mutations between the GEJ and stomach

**Figure 4:**
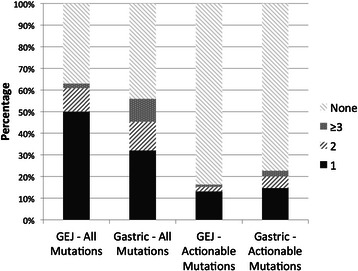
**Proportions of GEJ and gastric carcinomas with numbers of identified total and actionable mutations.** Solid dark areas in the columns represent cases with 1 mutation, dark diagonal lined areas represent cases with 2 mutations, and spotted areas represent cases with 3 or more mutations.

*TP53* mutations were identified in 39 of 92 (42%) of GEJ tumors, and in 20 of 75 (27%) gastric tumors (p = 0.036). When subdivided into the 3 subtypes suggested by Shah et al. [16], *TP53* mutations occurred more frequently in proximal nondiffuse cancers (44%) than in diffuse cancers (37%) and distal nondiffuse cancers (20%; p = 0.024). This classification also showed more frequent mutations in *KRAS* within distal nondiffuse cancers (12%) versus proximal nondiffuse (3%) and diffuse (0%) carcinomas (p = 0.12). No significant differences in mutation frequencies were present among the other individual genes in the panel. Two components of the Wnt pathway, *APC* and *CTNNB1*, were in aggregate mutated more frequently in gastric carcinomas than in GEJ tumors (16% vs. 3%, p = 0.006). Gastric carcinomas more frequently had mutations in 3 or more genes (11% vs. 2%, p = 0.044; Figure 4). No differences in the involvement of oncogenic pathways were noted between the two sites, based on mutational profiles.

### Potentially actionable mutations

Targeted therapies are available or in development for mutations occurring in the following genes: *AKT* [25], *BRAF* [26], *ERBB2* [27], *ERBB4* [28], *FGFR1* [29], *FGFR3* [30], *FLT3* [31,32], *IDH1* [33], *JAK3* [31], *KDR* [34,35], *KRAS* [36], *MET* [34], *PDGFRA* [37], *PIK3CA* [25], *PTEN* [25], *PTPN11* [38], *RET* [39], *SMO* [40]. Mutations in these genes were identified in 32 cases (19%), including 6 cases (4%) with 2 mutations and 3 cases (2%) with ≥3 mutations. The distribution of actionable mutations was not significantly different between GEJ and gastric carcinomas (p = 0.327; Figure 4).

### Prognostic significance of mutations

*ERBB4* mutations were associated with worse disease-free survival (p = 0.018), while there was a trend towards worse disease-free survival associated with mutations in *ABL1* (p = 0.063) and *JAK3* (p = 0.059). None of these mutations were prognostically significant after accounting for age, sex, Lauren subtype, stage, grade and margin status. Mutations in *BRAF* (p < 0.001), *FGFR3* (p < 0.001), *FLT3* (p < 0.001) were associated with worse overall survival on univariate analysis as a result of a single case with mutations in all three of these genes.). *BRAF* mutation remained prognostically significant after accounting for age, sex, Lauren subtype, stage, grade and margin status (p = 0.002).

### Comparison with TCGA data

**Figure 5:**
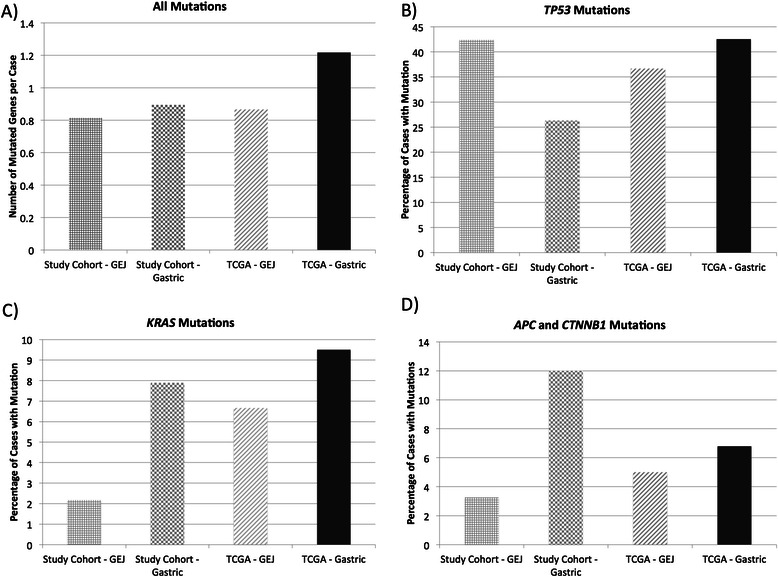
**Comparison of the frequency of mutations within hotspots identified in the study cohort using panel sequencing, compared to mutations identified using whole exome sequencing in the TCGA data. A)** Mutations across mutational hotspots in the 46 genes in the panel, **B)** mutations in *TP53*, **C)** mutations in *KRAS*, and **D)** mutations in the Wnt signaling components *APC* and *CTNNB1*.

When assessing the hotspot regions covered by the sequencing panel, the overall number of mutated genes per case was similar between the TCGA and study cohorts (p = 0.659), including when comparing either GEJ (p = 0.399) or gastric (p = 0.845) tumors only (Figure 5A). A trend towards more frequent cases with mutations in ≥3 genes in the stomach compared to the GEJ was also observed in the TCGA data (12% vs. 3%, p = 0.054). The overall frequency of *TP53* mutations was not different between the study cohort and the TCGA cohort (p = 0.230). No differences in *TP53*, *KRAS*, and *APC*/*CTNNB1* mutation rates between GEJ and gastric carcinomas were observed in the TCGA dataset (Figures 5B-D). The mutated genes in the TCGA data set are included in Additional file 7: Table S7. Regarding the mutations with possible prognostic significance identified in our cohort, there was a trend towards worse overall survival associated with *BRAF* mutations (p = 0.079), while no prognostic association was found in the TCGA cohort in association with mutations in *ERBB4*, *ABL1*, *JAK3*, *FLT3* or *FGFR3*.

## Discussion

This study aimed to probe the utility of panel sequencing in identifying single nucleotide changes in routinely processed gastric resection specimens, which could be used to guide targeted therapies. We secondarily sought to contrast GEJ and gastric carcinomas through targeted deep sequencing of a panel of 46 cancer-related genes, which revealed some differences at the genomic level that may reflect differing clinicopathologic profiles. Finally, we also sought to compare the frequencies of mutations obtained using this panel with results from whole exome sequencing in The Cancer Genome Atlas.

Adenocarcinomas of the gastrointestinal tract are molecularly heterogeneous and complex [41-44]. In gastric carcinoma, deep sequencing of single nucleotide polymorphism and RNA expression arrays have recently revealed abnormalities in several pathways including WNT, Hedgehog, cell cycling, DNA damage repair and the epithelial-to-mesenchymal transition [45]. The current use of multiple single gene tests is untenable given this complexity, particularly in the presence of a growing number of targeted therapies, constrained resources, and limited tissue availability. Thus, it is desirable to investigate multiple genes simultaneously. Panel sequencing has a sensitivity of close to 100% relative to conventional assays such as Sanger sequencing and PCR-based methods, as well as an ability to detect SNVs and INDELs at allele frequencies as low as 5% and 20%, respectively, in both FFPE [21,46-48] and cytology specimens [49-52]. Targeted panel sequencing can detect aberrations in cancer-related genes in early gastric cancers and precursors lesions [53], and its deep coverage could be particularly useful in gastric cancer by providing adequate results despite scant biopsy material and the admixture of tumor cells with desmoplasia and inflammatory cells.

Putative driver mutations were identified in a majority of GEJ and gastric carcinomas investigated in this study. By far the most frequently detected mutated gene was *TP53*, and these mutations have also been detected in early stage and precursor lesions using the same assay [53]. Multiple driver mutations were identified in several cases, reinforcing the idea that multiple genes need to be interrogated at once in genomically complex tumors such as gastric adenocarcinomas. A case with a mutation in *BRAF* (as well as FLT3 and FGFR3) was associated with poor overall survival on both univariate and multivariable analysis. This finding mirrors a trend observed in the TCGA data towards poor overall survival in *BRAF*-mutated tumours, suggesting that in some cases panel sequencing could have a prognostic role.

We were also able to detect potentially actionable mutations in approximately 20% of cases, which involved either genes or pathways where targeted therapies are available or in development. While this number would ideally be higher, our assay only covered certain hotspot regions of these genes, and did not account for copy number alterations that could also yield useful information. Further refinement of such panels to include a broader range of genes and gene segments will likely increase the proportion of cases in which mutations are identified. For example, although *TP53* mutations occur throughout the gene, the panel primarily covers exons 5–8, and some of the gene segments that were not sequenced are more frequently associated with loss of p53 on immunohistochemistry [54]. This fact may potentially explain both the differences in the rates of *TP53* mutations and patterns of immunohistochemical expression observed in the GEJ and stomach. Nevertheless, this study does demonstrate that single nucleotide variants can be identified from routine/archival pathology materials, and that with additional refinement panel sequencing may have a significant role in the future.

An unexpected result of the cancer hotspot panel sequencing approach was the identification of mutations in genes usually associated with non-epithelial malignancies, such as *IDH1* R132H/R132C, *JAK3* V722I, and *FLT3* A680V. The *IDH1* variants identified occur primarily in glial and hematologic malignancies, and result in altered cancer cell metabolism [55]. To the best of our knowledge, these cases constitute the first report of pathogenic *IDH1* mutations in gastric cancer. Recently *IDH1* mutations have been targeted [33], and mutation-specific treatments are currently the aim of a phase I clinical trial that includes cholantiocarcinomas (http://clinicaltrial.gov/ct2/show/NCT02073994). *FLT3* mutations occur in a third of cases of acute myelogenous leukemia [56], and the point mutation resulting in the A680V substitution has not been previously described in gastric cancer, while being observed occasionally in AML [57]. Similarly, activating *JAK3* mutations such as V722I have been identified in acute megakaryoblastic leukemia [58] and NK/T-cell lymphoma [31], and only in a few cases of gastric and breast cancer [59].

Epidemiologic and clinicopathologic differences exist between GEJ and gastric carcinomas [60-62]. GEJ carcinomas in this cohort were associated with younger age, different histotypes, and worse disease-free survival. As in other series, the rates of p53 overexpression were higher in the GEJ, as were the rates of *TP53* mutation [10,11], while Wnt abnormalities were more common in the gastric carcinomas [12]. In addition, more frequently there were mutations across ≥3 genes in gastric carcinomas, suggesting a higher mutational load and/or a bias towards genes included in the panel compared to GEJ lesions. Although the absence of differences in actionable mutations suggests that tumors in these sites can be considered together, the differences in *TP53* and Wnt component mutation rates support the recent push to use location to distinguish proximal and distal gastric carcinomas as separate entities. Based on gene expression data, Shah et al. recently suggested that gastric carcinomas be grouped into three different subtypes [16]. The detection of more frequent *KRAS* mutations within distal non-diffuse carcinomas in our dataset when using this subclassification further supports pathologic classification of gastric cancers based on location and histotype.

Overall, mutation frequencies within the targeted hotspots were detected at a similar rate as those observed with exome sequencing in the TCGA data, also suggesting that with appropriate design, panel sequencing could be a viable method for interrogating multiple genes with a single test. Cases with mutations in ≥3 genes were also more common in the stomach in this cohort. However, the differences in mutation rates in *TP53*, *KRAS*, and *APC*/*CTNNB1* between GEJ and gastric carcinomas were not observed within the TCGA cohort, even after comparing mutation frequencies within specific gastric locations. It is uncertain whether differences in case selection relating to etiology, geography or ethnicity could account for such differences, or whether differences in sequencing technology or bioinformatic analyses may also have contributed to these divergent observations. Further studies directly comparing the two approaches and comparing different patient populations will further enhance our understanding of GEJ and gastric carcinoma.

### Study limitations

Regarding case selection, in the presence of gastroesophageal reflux many of the landmarks used to delineate the stomach from the esophagus are destroyed. This study relied on the epicenter of the tumor being 5 cm from the gastroesophageal junction. However, we derived this classification from pathology reports and could not confirm the gross descriptions, nor did we subclassify tumours by Siewert type. Many of the tumours in this series may have in fact been esophageal in origin, and this could explain the similarities of the tumours with esophageal adenocarcinoma (e.g. worse prognosis and rates of *TP53* mutations). The patients’ family histories were not recorded for correlation, and the presence of gastric and GEJ cancer risk factors such as *Helicobacter* infection and Barrett esophagus were also not recorded. Sampling for sequencing and tissue microarray construction was limited, and intratumoral heterogeneity was not addressed. No germline DNA was available for comparison; as a result some somatic variants, which contribute to carcinogenesis but are present at low frequencies as single nucleotide polymorphisms, may have been omitted. In addition, we did not perform validation with Sanger sequencing or other methods. As such, we could not confirm the assay’s sensitivity and specificity on this series. The assay has been shown to be accurate in other studies and in our own laboratory. Further validation of this platform with Sanger sequencing or other methods would be required before this assay could be used clinically.

## Conclusions

GEJ and gastric tumors differ in several clinicopathologic respects, including the frequencies of mutations in certain caner-related genes. Tailoring treatment towards individual gastric cancer patients will require in-depth characterization of their tumors. This study shows that such characterization will derive information from both traditional clinicopathologic parameters such as tumor location, as well as from emerging molecular assays. Targeted panel sequencing is an approach that can be applied towards routine pathology material and can simultaneously yield information on several genes. Refinement of this approach may be a powerful tool for pathologists and clinicians in the future.

## Additional files

Additional file 1: Table S1. List of genes and sequences of primer pairs used for DNA amplicon library construction.
Additional file 2: Table S2. Clinicopathologic data for the 167 cases in this series, including site, stage, margin status, immunohistochemistry and survival.
Additional file 3: Table S3. Summary of the clinocopathologic variables in the cohort’s clinicopathologic variables within proximal non-diffuse, diffuse, and distal non-diffuse carcinomas.
Additional file 4: Table S4. Univariate and multivariable analyses of clinicopathologic variables associated with progression-free survival. Univariate values were computed via the log-rank test, and multivariable values were computed via Cox Proportional Hazard regression analysis using forward stepwise selection.
Additional file 5: Table S5. Univariate and multivariable analyses of clinicopathologic variables associated with overall survival. Univariate values were computed via the log-rank test, and multivariable values were computed via Cox Proportional Hazard regression analysis using forward stepwise selection.
Additional file 6: Table S6. List of variant calls detected in each case in this series.
Additional file 7: Table S7. List of mutated genes in the TCGA gastric cancer dataset within regions that were amplified and sequenced in this study.

## Abbreviations

GEJ: Gastroesophageal junction
FFPE: Formalin-fixed paraffin embedded
INDELs: Insertions/deletions
SNVs: Single nucleotide variants
