# Supplementary material for: Retrospective review using targeted deep sequencing reveals mutational differences between gastroesophageal junction and gastric carcinomas
Source: BMC Cancer. 2015 Feb 6;15:32. doi: 10.1186/s12885-015-1021-7 (PMC4322811; doi:10.1186/s12885-015-1021-7)
Supplement: Additional file 3: Table S3. — Summary of the clinocopathologic variables in the cohort’s clinicopathologic variables within proximal non-diffuse, diffuse, and distal non-diffuse carcinomas. [file 12885_2015_1021_MOESM3_ESM.docx]

*Table S3: Summary of the clinocopathologic variables in the cohort’s clinicopathologic variables within proximal non-diffuse, diffuse, and distal non-diffuse carcinomas.*

| **Clinicopathologic Variable** | **Proximal**  **Non-diffuse**  **(n = 77)** | **Diffuse**  **(n = 41)** | **Distal**  **Non-diffuse**  **(n = 49)** | **Overall**  **(n = 167)** | **p** |
| --- | --- | --- | --- | --- | --- |
| **Age (mean, years)** | 61.3 ± 9.7  [33-79] | 60.7 ± 10.8  [37-80] | 70.1 ± 10.4  [33-84] | 63.7 +/- 10.9 [33-84] | <0.001 |
| **Sex**  Male  Female | 59 (77)  18 (23) | 25 (61)  16 ( 39) | 37 (76)  12 (25) | 121 (73)  46 (27) | 0.165 |
| **Histologic Subtype (Lauren)**  Intestinal  Diffuse  Mixed | 65 (84)  0  12 (16) | 0  41 (100)  0 | 36 (74)  0  13 (27) | 101 (60)  41 (25)  25 (15) | <0.001 |
| **T-Stage**  1  2  3  4 | 5 (7)  22 (29)  42 (55)  8 (10) | 3 (7)  15 (37)  20 (49)  3 (7) | 5 (10)  22 (45)  20 (41)  2 (4) | 13 (8)  59 (35)  82 (49)  13 (8) | 0.478 |
| **N-Stage**  0  1  2  3 | 13 (17)  45 (58)  13 (17)  6 (8) | 10 (24)  18 (44)  9 (22)  4 (10) | 19 (39)  21 (43)  8 (16)  1 (2) | 42 (25)  84 (50)  30 (18)  11 (7) | 0.108 |
| **Grade**  Well Differentiated (G1)  Moderately Differentiated (G2)  Poorly Differentiated (G3) | 5 (7)  36 (47)  36 (47) | 0 (0)  0 (0)  41 (100) | 4 (8)  23 (47)  22 (45) | 10 (6)  64 (38)  93 (56) | 0.001 |
| **Resection Margin**  Uninvolved  Involved | 61 (79)  16 (21) | 36 (88)  5 (12) | 42 (86)  7 (14) | 139 (83)  28 (17) | 0.423 |
| **Her2 Amplification**  Absent  Present | 64 (83)  13 (17) | 39 (95)  2 (5) | 41 (84)  8 (16) | 144 (86)  23 (14) | 0.163 |
| **BAF250a (ARID1a) Expression**  Intact  Absent | 61 (79)  16 (21) | 29 (71)  12 (29) | 34 (69)  15 (31) | 124 (74)  43 (26) | 0.393 |
| **p53 expression**  0 – absent  1 – normal (1-60%)  2 - increased (>60%) | 25 (33)  14 (18)  38 (49) | 26 (63)  7 (17)  8 (20) | 28 (57)  10 (20)  11 (22) | 79 (47)  31 (19)  57 (34) | 0.002 |
| **Mismatch Repair Proteins**  Intact  Abnormal | 67 (87)  10 (13) | 29 (71)  12 (29) | 38 (78)  11 (22) | 135 (80)  33 (20) | 0.091 |
| **Number of Recurrences** | 48 (62) | 20 (49) | 21 (43) | 89 (53) | 0.082 |
| **Median Progression-Free Survival (Months)** | 12 | 15 | 18 | 15 |  |
| **Number of Deaths** | 58 (75) | 28 (63) | 31 (63) | 117 (70) | 0.340 |
| **Median Overall Survival (Months)** | 20.0 | 20.0 | 21.0 | 20.0 |  |
