# Supplementary material for: Retrospective review using targeted deep sequencing reveals mutational differences between gastroesophageal junction and gastric carcinomas
Source: BMC Cancer. 2015 Feb 6;15:32. doi: 10.1186/s12885-015-1021-7 (PMC4322811; doi:10.1186/s12885-015-1021-7)
Supplement: Additional file 4: Table S4. — Univariate and multivariable analyses of clinicopathologic variables associated with progression-free survival. Univariate values were computed via the log-rank test, and multivariable values were computed via Cox Proportional Hazard regression analysis using forward stepwise selection. [file 12885_2015_1021_MOESM4_ESM.docx]

*Table S4: Univariate and multivariable analyses of clinicopathologic variables associated with progression-free survival. Univariate values were computed via the log-rank test, and multivariable values were computed via Cox Proportional Hazard regression analysis using forward stepwise selection.*

|  | **Univariate Analysis** | | **Multivariable Analysis** | |
| --- | --- | --- | --- | --- |
| **Clinicopathologic Variable** | **Log-rank X^2^** | **p** | **HR (95% CI)** | **p** |
| Sex (F vs. M) | 2.76 | 0.097 | 0.60 (0.34-1.05) | 0.072 |
| Age | 0.59 (for age >65) | 0.443 | 1.02 (0.99-1.04) | 0.121 |
| Location (cardia vs non-cardia) | 10.04 | **0.002** | 2.30 (1.36-3.89) | **0.002** |
| Histologic Subtype  Diffuse vs. Intestinal  Mixed vs. Intestinal  Mixed vs. Diffuse | 3.30 | 0.192 | 1.19 (0.64 – 2.21)  0.79 (0.40 – 1.56)  1.51 (0.71 – 3.17) | 0.561 |
| AJCC Stage  II vs. I  III vs. I  III vs. II | 12.39 | **0.002** | 1.92 (0.82-4.49)  2.67 (1.04-6.88)  0.72 (0.42-1.23) | 0.120 |
| Grade  Grade 2 vs. Grade 1  Grade 3 vs. Grade 1  Grade 3 vs. Grade 2 | 1.85 | 0.396 | 2.86 (0.94-8.33)  1.78 (0.61-5.26)  1.59 (0.97-2.63) | 0.066 |
| Resection Margin Involvement | 7.23 | **0.007** | 1.92 (1.06-3.46) | **0.030** |
| Her2 Amplification | 2.79 | 0.095 | 1.31 (0.69-2.47) | 0.406 |
| Microsatellite Instability | 6.30 | **0.012** | 0.48 (0.23-0.99) | **0.048** |
| BAF250a Loss | 0.03 | 0.861 | 1.40 (0.83-2.35) | 0.205 |

*df: degrees of freedom; p: p-value; HR: Hazard Ratio; 95% CI: 95% upper and lower confidence intervals.*
