# Supplementary material for: Retrospective review using targeted deep sequencing reveals mutational differences between gastroesophageal junction and gastric carcinomas
Source: BMC Cancer. 2015 Feb 6;15:32. doi: 10.1186/s12885-015-1021-7 (PMC4322811; doi:10.1186/s12885-015-1021-7)
Supplement: Additional file 5: Table S5. — Univariate and multivariable analyses of clinicopathologic variables associated with overall survival. Univariate values were computed via the log-rank test, and multivariable values were computed via Cox Proportional Hazard regression analysis using forward stepwise selection. [file 12885_2015_1021_MOESM5_ESM.docx]

*Table S5: Univariate and multivariable analyses of clinicopathologic variables associated with overall survival. Univariate values were computed via the log-rank test, and multivariable values were computed via Cox Proportional Hazard regression analysis using forward stepwise selection.*

|  | **Univariate Analysis** | | **Multivariable Analysis** | |
| --- | --- | --- | --- | --- |
| **Clinicopathologic Variable** | **Log-rank X^2^** | **p** | **HR (95% CI)** | **p** |
| Sex (F vs. M) | 0.08 | 0.784 | 0.87 (0.55-1.38) | 0.873 |
| Age | 0.00 (for age >65) | 0.976 | 1.03 (1.00-1.05) | **0.016** |
| Location (cardia vs non-cardia) | 1.47 | 0.225 | 1.43 (0.91-2.26) | 0.120 |
| Histologic Subtype  Diffuse vs. Intestinal  Mixed vs. Intestinal  Mixed vs. Diffuse | 1.05 | 0.591 | 1.17 (0.67-2.02)  0.83 (.046-1.52)  1.40 (0.73-2.69) | 0.597 |
| AJCC Stage  II vs. I  III vs. I  III vs. II | 10.62 | **0.005** | 1.49 (0.79-2.82)  2.39 (1.13-5.05)  1.61 (0.99-2.56) | 0.053 |
| Grade  Grade 2 vs. Grade 1  Grade 3 vs. Grade 1  Grade 3 vs. Grade 2 | 6.96 | **0.03** | 3.45 (1.47-8.33)  2.50 (1.04-5.88)  1.41 (0.89-2.22) | **0.012** |
| Resection Margin Involvement | 6.04 | **0.014** | 1.83 (1.09-3.09) | **0.023** |
| Her2 Amplification | 1.96 | 0.161 | 1.09 (0.62-1.91) | 0.777 |
| Microsatellite Instability | 2.61 | 0.106 | 0.72 (0.39-1.34) | 0.297 |
| BAF250a Loss | 0.01 | 0.916 | 1.05 (0.65-1.71) | 0.836 |

*df: degrees of freedom; p: p-value; HR: Hazard Ratio; 95% CI: 95% upper and lower confidence intervals.*
